# Occult metastases detection and impact on management in renal cell carcinoma imaged with [^89^Zr]Zr-girentuximab

**DOI:** 10.1007/s00259-026-08105-9

**Published:** 2026-08-05

**Authors:** Alexander Weissensee, Christian Rothermundt, Tobias Peres, Savo Matijasevic, Thiago Lima, Hannes Grünig, Andrea Zander, Tatjana Leike, Klaus Strobel

**Affiliations:** 1 Department of Nuclear Medicine and Radiology, Cantonal Hospital Lucerne, University Teaching and Research Hospital, University of Lucerne, Luzern, Switzerland; 2 Department of Medical Oncology and Cancer Centre, Cantonal Hospital Lucerne, University Teaching and Research Hospital, University of Lucerne, Luzern, Switzerland; 3 Department of Medical Oncology and Hematology, HOCH Health Ostschweiz, Kantonsspital St. Gallen (KSSG), St. Gallen, Switzerland

In this 46-year-old man, MRI performed three years after resection of a clear cell renal cell carcinoma (ccRCC) and adjuvant pembrolizumab revealed new suspicious lesions in the right kidney (A1) and paraspinal muscles (A2) (Fig. 1). Biopsy of the paraspinal lesion confirmed metastatic ccRCC. [^89^Zr]Zr-girentuximab PET/CT was performed to optimize staging and guide treatment planning. We administered 38 MBq of [^89^Zr]Zr-girentuximab and scanned the patient 5 days later. During this outpatient procedure we instructed the patient to follow radioprotection measures comparable to patients discharged after radionuclide therapy. The two known kidney and muscle lesions showed intense tracer uptake (B1). Additionally, [^89^Zr]Zr-girentuximab PET/CT detected three metastases that were occult on MRI and low-dose CT: a second renal lesion (B2, A3), a small thoracic wall soft tissue nodule (B3, C1), and an axillary lymph node (B4, C2). All lesions showed remarkable signal-to-background ratios and conspicuity, as exemplified by an SUV_max_ of 48.0 for the 3 × 3 mm soft tissue lesion. Histopathology confirmed the axillary lymph node as a ccRCC metastasis. The other lesions were characterized on imaging alone and not confirmed on pathology. The precise delineation of disease had therapeutic impact by guiding local radiotherapy and cryoablation for all metastases [1–3]. [^89^Zr]Zr-girentuximab PET/CT is a highly accurate, non-invasive method to identify and characterize ccRCC in patients with indeterminate renal masses ≤ 7 cm, achieving sensitivities and specificities of 86% and 87%, respectively [4]. Current guidelines recognize CAIX-targeted girentuximab PET as an emerging tool for risk stratification of renal masses [5]. Notably, extremely small lesions were detected with high lesion-to-background contrast, underscoring the sensitivity of CAIX-targeted imaging. The mean whole body effective dose of 0.57 ± 0.08 mSv/MBq for this tracer is relatively high [6]. Denoising approaches and new sensitive PET scanners, especially long-axial-field-of-view (LAFOV) PET systems, might allow substantial dose reduction while maintaining image quality. The long physical half-life of [^89^Zr] requires imaging planning approx. 5 days after tracer administration. This case highlights the value of [^89^Zr]Zr-girentuximab PET/CT for accurate staging and detection of occult metastases in ccRCC, with direct implications for treatment planning.

**Fig. 1:**
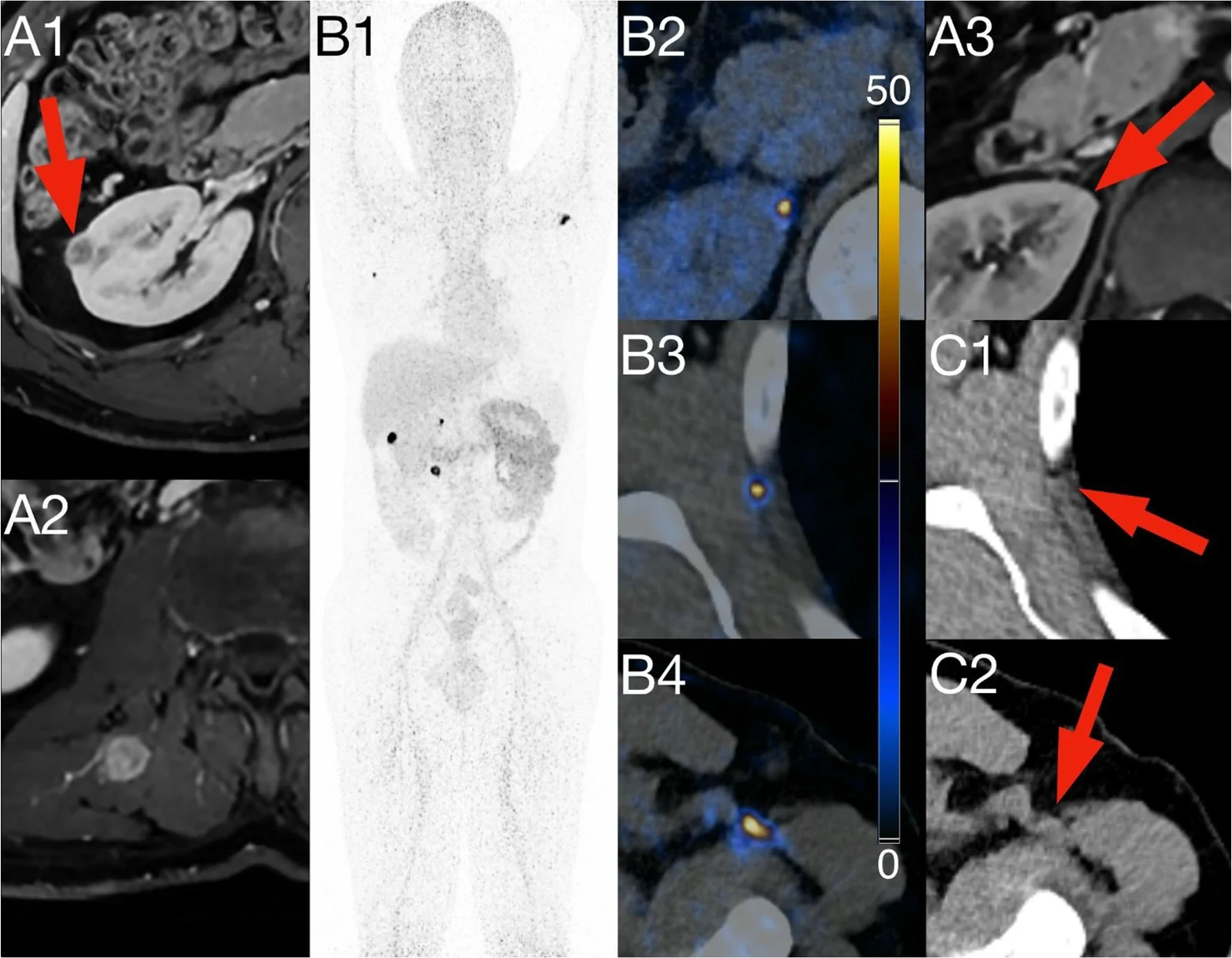
Image Panel consisting of MRI (A1-3), PET maximum-intensity projection (MIP; B1), fused PET/CT (B2-4), and low-dose CT images (C1-C2). Red arrows indicate the morphological correlates of the intensely [^89^Zr]Zr-girentuximab-avid lesions. A2 depicts a contrast-enhancing lesion in the right paraspinal musculature, which also showed high uptake of [^89^Zr]Zr-girentuximab

## Data Availability

Data sharing is not applicable to this article as no datasets were generated or analyzed during the current study.
